# Rare cas de tuberculose multifocale au Burkina Faso chez un sujet drépanocytaire SC avec une localisation atypique : l'articulation sterno-claviculaire

**DOI:** 10.48327/mtsi.v4i3.2024.506

**Published:** 2024-08-28

**Authors:** Yannick Laurent Tchenadoyo BAYALA, Ismaël AYOUBA TINNI, Fulgence KABORÉ, Marcellin BONKOUNGOU, Wendlassida Joëlle Stéphanie ZABSONRÉ, Dieu-Donné OUEDRAOGO

**Affiliations:** Service de rhumatologie du Centre hospitalier universitaire de Bogodogo, Ouagadougou, Burkina Faso

**Keywords:** *Mycobacterium tuberculosis*, Tuberculose multifocale, Articulation sterno-claviculaire, Drépanocytose, Ouagadougou, Burkina Faso, Afrique subsaharienne, *Mycobacterium tuberculosis*, Multifocal tuberculosis, Sternoclavicular joint, Sickle cell disease, Ouagadougou, Burkina Faso, Sub-Saharan Africa

## Abstract

**Introduction:**

La tuberculose et la drépanocytose sont fréquentes en Afrique au sud du Sahara. La tuberculose multifocale, plus courante chez les immunodéprimés, est rare chez les drépanocytaires SC, surtout lorsqu'elle est localisée à l'articulation sterno-claviculaire.

**Observation:**

Patient de 44 ans drépanocytaire, noir africain, d'origine burkinabè, sans autres antécédents pathologiques, reçu pour une lombalgie chronique inflammatoire associée à une gonalgie inflammatoire droite, et une toux grasse. Cette symptomatologie évoluerait depuis sept mois dans un contexte fébrile et d'altération de l’état général. L'examen montrait une oligoarthrite de l'articulation sterno-claviculaire droite et du genou gauche, associée à un syndrome de condensation pulmonaire et d’épanchement pleural, un abcès froid au niveau de l'articulation sterno-claviculaire droite et une adénopathie inguinale droite fistulisée et purulente. La biologie montrait un syndrome inflammatoire. Le test GeneXpert était positif dans les crachats, sans résistance à la rifampicine. L'intradermo-réaction à la tuberculine était positive. La tomodensitométrie thoracique montrait une ostéoarthrite sterno-claviculaire droite et celle du rachis lombaire une spondylodiscite L3-L4. La radiographie standard du genou gauche objectivait des signes en faveur d'une arthrite. Le diagnostic de tuberculose avec atteinte osseuse multifocale, pleuropulmonaire et ganglionnaire a été retenu. Le patient a été mis sous antalgique usuel et sous antituberculeux. L’évolution a été favorable.

**Conclusion:**

La tuberculose multifocale peut affecter les drépanocytaires SC, nécessitant une vigilance pour prévenir les complications, notamment les localisations articulaires rares comme l'articulation sterno-claviculaire.

## Abstract (en)

**Introduction:**

Tuberculosis and sickle cell disease are common in sub-Saharan Africa. Multifocal tuberculosis, more common in immunocompromised patients, is rare in SC sickle cell patients, especially when localized to the sternoclavicular joint.

**Case report:**

A 44-year-old male sickle cell patient, black African, of Burkina Faso origin, with no other pathological history, presented with chronic inflammatory low back pain associated with right-sided inflammatory gonalgia and hacking cough. This symptomatology had been developing for 7 months in the context of fever and altered general condition. Examination revealed oligoarthritis of the right sternoclavicular joint and the left knee associated with pulmonary condensation syndrome and pleural effusion, a cold abscess in the right sternoclavicular joint, and a purulent fistulized right inguinal adenopathy. Biology revealed a biologic inflammatory syndrome. The GeneXpert test was positive in sputum, with no resistance to rifampin. Intradermal tuberculin test was positive. Chest CT revealed right sternoclavicular osteoarthritis and lumbar CT revealed L3-L4 spondylodiscitis. A standard radiograph of the left knee showed signs of arthritis. The diagnosis was tuberculosis with multifocal bone, pleural, and lymph node involvement. The patient was started on standard analgesics and antituberculosis drugs. The course was favorable.

**Conclusion:**

Multifocal tuberculosis may affect SC sickle cell patients and requires vigilance to prevent complications, especially in rare articular localizations such as the sternoclavicular joint.

## Introduction

La tuberculose est une maladie courante dans les pays en voie de développement, plus fréquente chez les sujets immunodéprimés et exceptionnellement décrite chez les sujets drépanocytaires [4]. Elle peut toucher la quasi-totalité des organes. Cependant, les formes multifocales sont rares et représentent 9 à 10 % des localisations extrapulmonaires [11]. La localisation de l'articulation sterno-claviculaire est extrêmement rare et souvent mal diagnostiquée en raison de sa rareté. Elle constitue 1 à 2 % des localisations osseuses de la tuberculose [6]. Nous décrivons à travers cette observation un cas rare de tuberculose multifocale avec une localisation sterno-claviculaire chez un sujet drépanocytaire majeur SC.

## Observation

**Figure 1:**
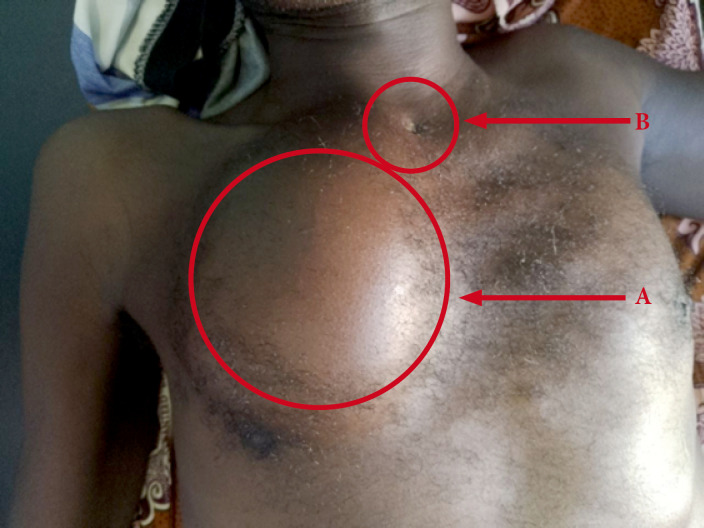
Tuméfaction (A) en regard de l'articulation sterno-claviculaire fistulisée (B) laissant sourdre du pus

**Figure 2:**
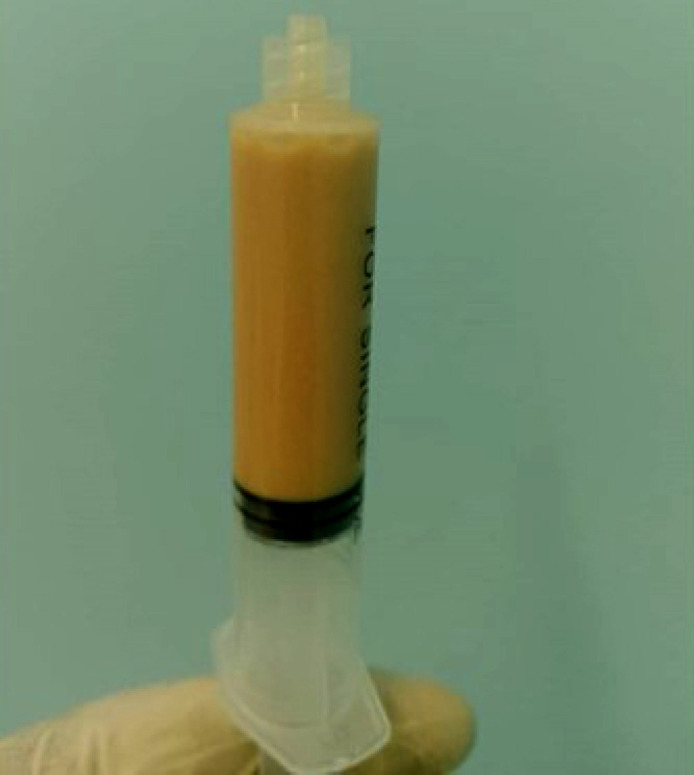
Liquide articulaire purulent issu de la ponction du genou gauche

**Figure 3:**
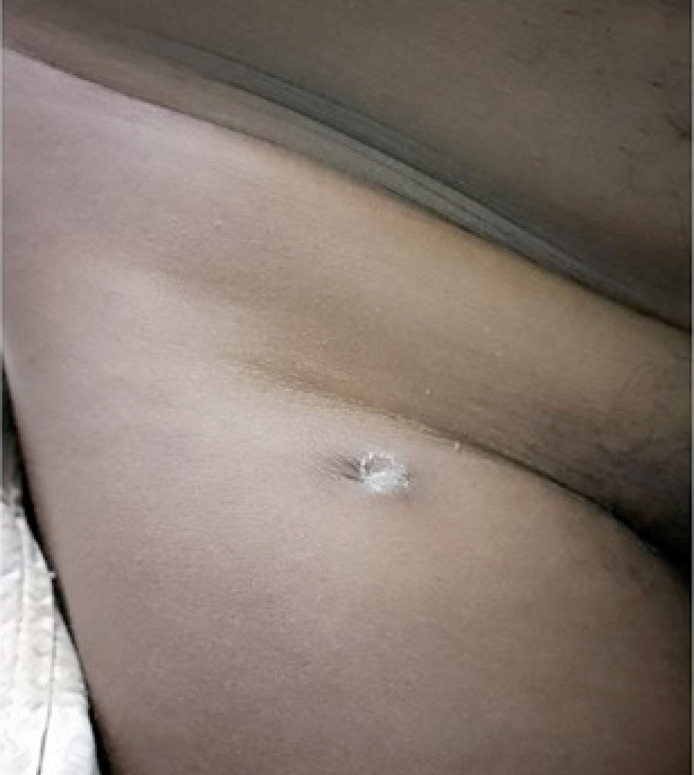
Adénopathie inguinale droite fistulisée laissant sourdre du pus

M. OI est un patient de 44 ans noir africain, d'origine burkinabè. Il est tabagique à 10 paquetsannée et drépanocytaire SC, suivi depuis une dizaine d'années et sous acide folique à 5 mg par jour. Il n'a pas de notion de contage tuberculeux, ni d'antécédents pathologiques particuliers. Il a été reçu au service de rhumatologie pour une lombalgie chronique inflammatoire associée à une gonalgie gauche inflammatoire évoluant depuis environ sept mois. Il aurait fait un traitement à base de décoction buvable sans effet sur la symptomatologie. À cette symptomatologie se serait associée une toux grasse dans un contexte de fièvre vespérale, d'asthénie physique modérée, d'anorexie et d'amaigrissement important. À l'examen physique, on notait une anémie clinique non décompensée, un syndrome rachidien et un psoïtis bilatéral. L'examen de l’épaule droite notait une tuméfaction molle et purulente de 4x5 cm en regard de l'articulation sterno-claviculaire (Fig. 1) et une mobilité normale de l’épaule droite. L'examen du genou a retrouvé une monoarthrite dont la ponction a révélé un liquide purulent et fluide (Fig. 2). L'examen de l'appareil respiratoire a montré un syndrome de condensation dans le champ pulmonaire gauche et un épanchement pleural purulent à droite. Une adénopathie inguinale droite avec du pus était notée (Fig. 3). L'examen des autres articulations et autres organes était sans particularité. À la biologie, on notait un syndrome inflammatoire biologique avec une anémie microcytaire hypochrome à 8,1 g/dl et une protéine C réactive (CRP) à 98 mg/l, pas d'hyperleucocytose.

**Figure 4:**
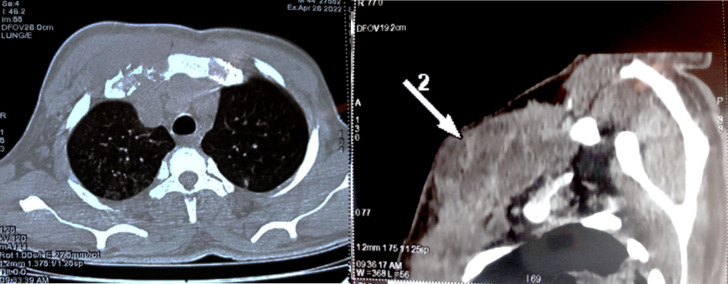
TDM thoracique objectivant une ostéoarthrite de l'articulation sterno-claviculaire droite

**Figure 5:**
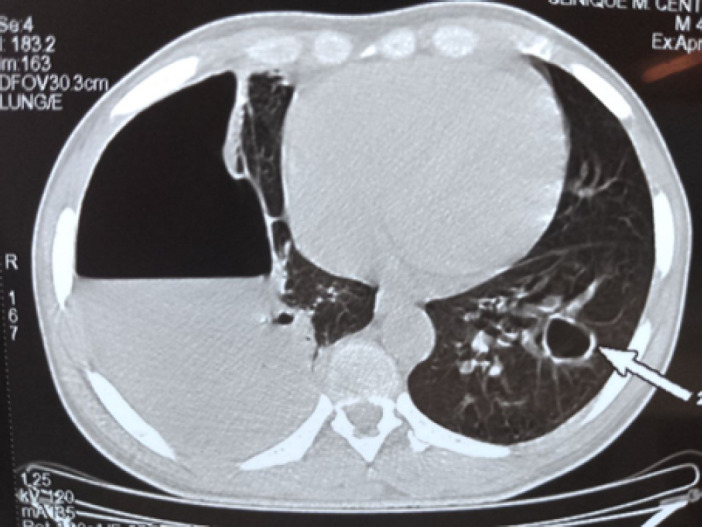
TDM thoracique objectivant un pyothorax droit et une caverne tuberculeuse au niveau du parenchyme pulmonaire gauche

**Figure 6:**
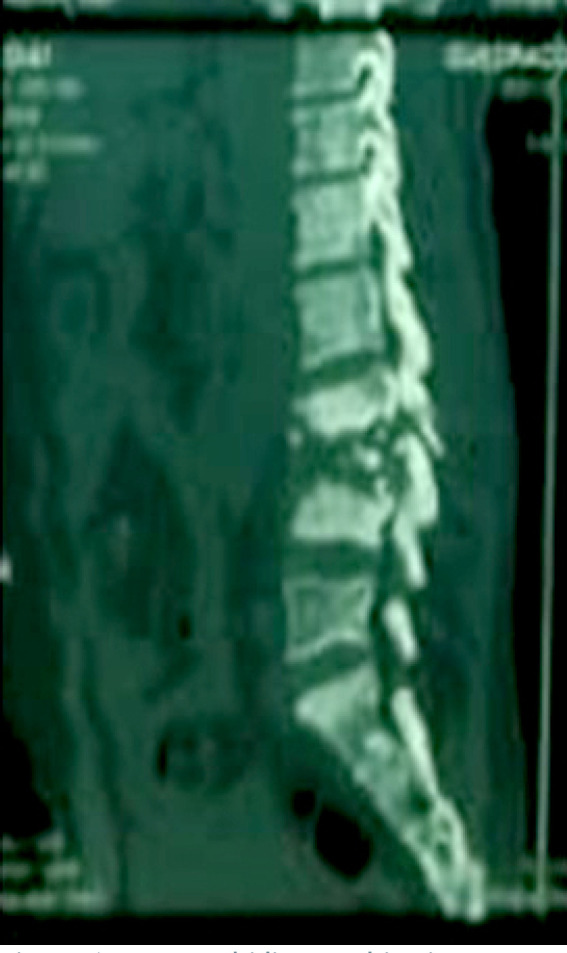
TDM rachidienne objectivant une spondylodiscite L3-L4

Le bilan rénal et hépatique était sans particularité. La sérologie rétrovirale au VIH était négative. L'intradermo réaction (IDR) à la tuberculine était positive à 18 mm. Le test GeneXpert MTB/RIF isolait dans des crachats le complexe *Mycobacterium tuberculosis* sensible à la rifampicine. L'examen cytobactériologique, le test GeneXpert MTB/RIF ainsi que la recherche de bacilles acido-alcoolo résistants (BAAR) dans le pus issu des différents sites (plèvre, genou, adénopathie inguinale et abcès froid sterno-claviculaire) n'ont pas trouvé de germe. À l'imagerie, la tomodensitométrie thoracique montrait une ostéoarthrite sternoclaviculaire droite (Fig. 4) avec une extension musculaire et médiastinale antérieure associée à un pyothorax et un abcès pulmonaire droit (Fig. 5). La tomodensitométrie (TDM) du rachis lombaire montrait une spondylodiscite L3-L4 avec des abcès calcifiants des muscles psoas et paravertébraux (Fig. 6). La radiographie standard du genou gauche objectivait des signes en faveur d'une arthrite. Le diagnostic de tuberculose avec atteinte osseuse multifocale, pleuropulmonaire et ganglionnaire a été retenu devant ce tableau clinique et biologique. Le patient a été mis sous rifampicine 600 mg par jour, isoniazide 300 mg par jour, pyrazinamide 2 000 mg par jour et éthambutol à 1 600 mg par jour pendant deux mois. Les mêmes doses de rifampicine et d'isoniazide ont été poursuivies durant 10 mois. Le patient a aussi bénéficié d'un drainage thoracique du pyothorax au bloc opératoire et d'un lavage articulaire du genou gauche, ainsi que d'une ponction évacuatrice de l'abcès froid en regard de l'articulation sterno-claviculaire. Enfin, des soins locaux à base d'antiseptique ont été appliqués à la fistule de l'adénopathie inguinale et de l'abcès froid jusqu’à cicatrisation complète. Le traitement d'acide folique dans le cadre de la drépanocytose a été poursuivi. L’évolution immédiate fut marquée par une amélioration clinique et biologique avec disparition du syndrome inflammatoire et réduction de la fièvre. L’évolution à trois mois s'est caractérisée par une amélioration du psoïtisme et des lombalgies, et une prise de poids estimée à 5 %.

## Discussion

La tuberculose est un sujet de santé publique, notamment dans les régions à forte charge de morbidité comme l'Afrique subsaharienne [11]. Des facteurs de risque d'immunodépression, tels que l'infection par le VIH, le diabète sucré, l'utilisation de corticoïdes, l'alcoolisme, l'insuffisance rénale ou les cancers, sont associés à la survenue de la tuberculose [5]. Par contre, la drépanocytose ne fait pas partie de ces terrains favorisants et la tuberculose chez les drépanocytaires est peu documentée [8]. En Inde, Kumar *et al.* trouvaient seulement cinq cas de tuberculose chez 166 drépanocytaires hospitalisés pour infection bactérienne [6]. Dans la cohorte française étudiée par Lionnet *et al.,* aucun patient ne présentait d'atteinte multifocale [8]. La tuberculose multifocale chez un sujet drépanocytaire avec une localisation de l'articulation sterno-claviculaire est une présentation rare, et à notre connaissance, notre cas est le seul rapporté à ce jour.

L'arthrite tuberculeuse de l'articulation sternoclaviculaire représente moins de 0,5 % des infections osseuses et articulaires. Les articulations périphériques portantes sont généralement les plus touchées [6]. Selon Booth *et al.,* les mécanismes d'immunodépression chez les drépanocytaires, en particulier ceux liés à l'asplénie fonctionnelle, favorisent les infections par des bactéries encapsulées [3]. Il est établi que la tuberculose n'est pas fréquente chez les drépanocytaires. L'association de l'hémoglobinose SC et de la tuberculose multifocale, comme décrite dans notre cas, pourrait être purement fortuite. Les germes connus pour donner une infection pour les drépanocytaires, tels que *Salmonella* spp., *Streptococcus pneumoniae*, *Haemophilus influenzae*, *Staphylococcus aureus* ont été évoqués en premier et rejetés par la suite [10]. La présence inhabituelle de *M. tuberculosis* dans ce contexte clinique mérite donc une attention particulière, mais elle ne doit pas éclipser la nécessité d'envisager d'autres agents plus fréquents.

Dans les formes monoarticulaires, la littérature rapporte fréquemment une tuméfaction et des douleurs à l’épaule accompagnées d'une limitation de sa mobilité, principal motif deconsultation [5]. L’évolution de la tuberculose sternoclaviculaire est habituellement fruste avec une douleur minime, contrairement à ce que l'on observe dans d'autres types d'arthrite septique [1]. Cependant, chez notre patient, la toux liée à l'atteinte pulmonaire était également prédominante. Nous avons exclu les autres étiologies d'arthrite sterno-claviculaire chez un drépanocytaire, à savoir l'ostéonécrose de l'extrémité médiale de la clavicule, l'arthrose, le syndrome de Tietze, l'hyperostose sterno-claviculaire et les rhumatismes inflammatoires chroniques [9]. La cause infectieuse était la plus probable devant la présence des abcès. Les germes les plus souvent rencontrés dans les arthrites infectieuses de l'articulation sterno-claviculaire sont S. *aureus* dans 49 % des cas, *Pseudomonas aeruginosa* dans 10 % des cas, *M. tuberculosis* ne représentant que 3 % des cas [7].

L'origine de la tuberculose ostéoarticulaire est le plus souvent hématogène et provient d'un foyer pulmonaire [7]. La tuberculose sterno-claviculaire est peu fréquente, car la circulation sanguine dans cette articulation provient d'une arcade de vaisseaux sanguins située à l'intérieur de l'artère mammaire interne [6]. Chez notre patient, une diffusion conjointe d'une tuberculose pulmonaire apicale vers l'articulation sterno-claviculaire peut être envisagée.

Quelques facteurs immunitaires chez les drépanocytaires peuvent expliquer leur taux bas de tuberculose. Les drépanocytaires présentent une stimulation constante des macrophages alvéolaires et une production élevée de cytokines locales [10]. Cette condition pro-inflammatoire habituellement perçue comme néfaste, car responsable de syndrome douloureux thoracique et d'hyperréactivité bronchique, peut offrir aux drépanocytaires une protection spécifique contre la tuberculose [2]. La fréquence de la tuberculose extrapulmonaire tend à augmenter si la réponse immunitaire cellulaire est compromise, comme c'est le cas dans l'infection par le VIH [11]. Cependant, ni l'infection à VIH, ni aucun autre facteur d'immunodépression n’étaient trouvés chez notre patient.

La tuberculose de l'articulation sterno-claviculaire est difficile à diagnostiquer, ce qui peut retarder le traitement. La tuberculose ostéoarticulaire en général est une maladie pauci-bacillaire : un test de l'IDR peut être négatif dans de nombreux cas et la plupart des patients ont également une radiographie normale. L'histologie et la culture des biopsies sont les normes de référence pour le diagnostic, fournissant des résultats positifs dans plus de 90 % des cas. Le test de GeneXpert est un bon outil, car il peut détecter même des traces d'ADN de *M. tuberculosis.* Concernant l'articulation sterno-claviculaire, la TDM peut s'avérer utile en visualisant les signes en faveur d'une arthrite avec atteinte des parties molles. La présence d'abcès calcifiants au niveau des muscles paravertébraux et du psoas est quasi pathognomonique de l'origine tuberculeuse. Les modalités radiologiques et d'imagerie sont complémentaires, l'IRM étant la meilleure technique pour la détection précoce et le diagnostic de la tuberculose de l'articulation sterno-claviculaire [11].

Le caractère multifocal de la tuberculose chez notre patient peut s'expliquer par le retard de consultation d'un centre de santé. Ce retard est fréquent dans le contexte tropical et africain. Les patients ont souvent recours en premier lieu aux tradipraticiens. A ce retard s'ajoute celui du diagnostic lié au manque de plateau technique et celui de l'accès aux traitements. Ces retards peuvent altérer le pronostic [4]. Le pronostic vital de notre patient était réservé surtout du fait de l'atteinte pleuropulmonaire.

## Conclusion

La tuberculose multifocale est une forme grave touchant habituellement les immunodéprimés ayant déjà une localisation pulmonaire. Elle est rare chez les sujets drépanocytaires. Elle ne doit pas être méconnue. La localisation articulaire sterno-claviculaire est exceptionnelle. Il est nécessaire de faire systématiquement un bilan exhaustif de dissémination du germe de la tuberculose chez les sujets drépanocytaires pour en assurer une meilleure prise en charge.

## Consentement du patient

Nous avons obtenu le consentement du patient pour la publication de cet article

## Contribution des auteurs

Bayala Yannick Laurent Tchenadoyo : rédaction, conception, discussion

Ayouba Tinni Ismael : conception, discussion

Kaboré Fulgence, Bonkoungou Marcellin : validation

Zabsonré/Tiendrebeogo Wendlassida Joëlle Stéphanie, Ouedraogo Dieu-Donné : supervision.

## Conflit d'intérêt

Les auteurs ne rapportent aucun conflit d'intérêt.
