# Supplementary material for: Rare case of multifocal tuberculosis in Burkina Faso in a sickle cell SC patient with an atypical location: the sternoclavicular joint
Source: Med Trop Sante Int. 2024 Aug 28;4(3):mtsi.v4i3.2024.506. [Article in French] doi: 10.48327/mtsi.v4i3.2024.506 (PMC11809065; doi:10.48327/mtsi.v4i3.2024.506)
Supplement: Related texts

# Research article: Rare case of multifocal tuberculosis in Burkina Faso in a sickle cell SC patient with an atypical location: the sternoclavicular joint

## Introduction

Tuberculosis is a common disease in developing countries, more common in immunocompromised individuals, and exceptionally described in sickle cell patients [4]. It can affect almost all organs. However, multifocal forms are rare, accounting for 9-10% of extrapulmonary localizations [11]. Sternoclavicular joint involvement is extremely rare and often misdiagnosed due to its rarity. It accounts for 1 to 2% of tuberculosis bone localizations [6]. In this case report, we describe a rare case of multifocal tuberculosis with sternoclavicular localization in a patient with sickle cell anemia major SC.

## Observation

Mr. OI is a 44-year-old black African patient from Burkina Faso. He is a 10 pack-year smoker and sickle cell disease patient who has been followed for about ten years and takes 5 mg of folic acid daily. He has no history of tuberculosis and no particular pathological antecedents. He was referred to the rheumatology department for chronic inflammatory low back pain associated with inflammatory left gonalgia of approximately seven months’ duration. He had been treated with an oral decoction with no effect on his symptoms. This symptomatology was associated with a hacking cough in the context of evening fever, moderate physical asthenia, anorexia, and significant weight loss. Physical examination revealed clinically undercompensated anemia, spinal syndrome, and bilateral psoitis. Examination of the right shoulder revealed a soft, purulent swelling measuring 4x5 cm opposite the sternoclavicular joint (Fig. 1) and normal range of motion of the right shoulder. Examination of the knee revealed monoarthritis, the puncture of which revealed a purulent fluid (Fig. 2). Examination of the respiratory system showed condensation syndrome in the left lung field and a purulent pleural effusion on the right. A right groin adenopathy with pus was noted (Fig. 3). Examination of other joints and organs was unremarkable. Biology revealed a biologic inflammatory syndrome with microcytic hypochromic anemia at 8.1 g/dL and C-reactive protein (CRP) at 98 mg/dL, without hyperleukocytosis. Renal and liver function tests were normal. HIV retroviral serology was negative.

**Figure 1:** Swelling (A) in relation to the fistulised sternoclavicular joint (B) leaking pus

**Figure 2:** Purulent joint fluid from left knee puncture

**Figure 3:** Fistulised right inguinal adenopathy leaking pus

The tuberculin skin test (TST) was positive at 18 mm. The GeneXpert MTB/RIF Test isolated rifampicin-sensitive *Mycobacterium tuberculosis* complex from sputum. Cytobacteriology, the GeneXpert MTB/RIF test, and acid-fast bacilli (AFB) testing of pus from various sites (pleural, knee, inguinal adenopathy, and sternoclavicular cold abscess) were negative for organisms. On imaging, the thoracic CT scan showed right sternoclavicular osteoarthritis (Fig. 4) with muscular and anterior mediastinal extension associated with pyothorax and right lung abscess (Fig. 5). Computed tomography (CT) of the lumbar spine showed L3-L4 spondylodiscitis with calcifying abscesses of the psoas and paravertebral muscles (Fig. 6). Standard radiographs of the left knee showed signs of arthritis. Based on this clinical and biological picture, a diagnosis of tuberculosis with multifocal bone, pleuropulmonary, and lymph node involvement was made. The patient was started on rifampicin 600 mg daily, isoniazid 300 mg daily, pyrazinamide 2,000 mg daily, and ethambutol 1,600 mg daily for two months. The same doses of rifampicin and isoniazid were continued for 10 months. The patient also underwent thoracic drainage of the pyothorax in the operating room, joint lavage of the left knee, and evacuation of the cold abscess above the sternoclavicular joint. Finally, the inguinal adenopathy fistula and cold abscess were treated with local antiseptics until complete healing. Folic acid treatment for sickle cell disease was continued. The immediate course was characterized by clinical and biological improvement, with disappearance of the inflammatory syndrome and reduction of fever. After three months, psoitis and low back pain improved, and weight gain was estimated at 5%.

**Figure 4:** Chest CT scan showing osteoarthritis of the right sternoclavicular joint

**Figure 5:** Chest CT scan showing a right pyothorax and a tuberculous cavern in the left lung parenchyma

**Figure 6:** Lumbar spine CT scan showing L3-L4 spondylodiscitis

## Discussion

Tuberculosis is a public health problem, especially in high-burden regions such as sub-Saharan Africa [11]. Risk factors for immunosuppression such as HIV infection, diabetes mellitus, corticosteroid use, alcoholism, renal failure or cancer are associated with the occurrence of tuberculosis [5]. However, sickle cell disease is not one of these conditions, and tuberculosis in sickle cell patients is poorly documented [8]. In India, Kumar *et al.* found only five cases of tuberculosis among 166 sickle cell patients hospitalized for bacterial infection [6]. In the French cohort studied by Lionnet *et al.,* no patient presented with multifocal involvement [8]. Multifocal tuberculosis in a sickle cell patient with sternoclavicular joint involvement is a rare presentation, and to our knowledge, our case is the only one reported to date. Tuberculous arthritis of the sternoclavicular joint accounts for less than 0.5% of bone and joint infections. Peripheral weight-bearing joints tend to be most affected [6]. According to Booth *et al.,* the mechanisms of immunosuppression in sickle cell disease, particularly those associated with functional asplenia, favor infection by encapsulated bacteria [3]. Tuberculosis is not common in sickle cell disease. The association of SC hemoglobinopathy and multifocal tuberculosis, as described in our case, could be purely coincidental. Germs known to cause infections in sickle cell disease, such as *Salmonella* spp., *Streptococcus pneumoniae, Haemophilus influenzae,* and *Staphylococcus aureus,* were initially proposed and subsequently rejected [10]. The unusual presence of *M. tuberculosis* in this clinical context therefore deserves special attention, but should not overshadow the need to consider other more common pathogens. In monoarticular forms, swelling and pain in the shoulder are often reported in the literature and are the main reason for consultation [5]. The course of sternoclavicular tuberculosis is usually mild, with minimal pain, in contrast to other types of septic arthritis [1]. However, our patient also had a predominant cough associated with pulmonary involvement. We excluded other etiologies of sternoclavicular arthritis in a sickle cell patient, namely osteonecrosis of the medial end of the clavicle, osteoarthritis, Tietze's syndrome, sternoclavicular hyperostosis, and chronic inflammatory rheumatism [9]. An infectious cause was most likely due to the presence of abscesses. The most common pathogens in infectious arthritis of the sternoclavicular joint were *S. aureus* in 49% of cases, *Pseudomonas aeruginosa* in 10%, and *M. tuberculosis* in only 3% of cases [7].

The origin of osteoarticular tuberculosis is most often hematogenous, originating from a pulmonary focus [7]. Sternoclavicular tuberculosis is rare because the blood supply to this joint originates from an arcade of vessels within the internal mammary artery [6]. In our patient, joint spread of apical pulmonary tuberculosis to the sternoclavicular joint can be considered.

Some immune factors in sickle cell patients may explain their low rate of tuberculosis. Sickle cell patients show a constant stimulation of alveolar macrophages and increased local cytokine production [10]. This pro-inflammatory state, usually considered detrimental because it is responsible for chest pain syndrome and bronchial hyperreactivity, may provide specific protection against tuberculosis in sickle cell patients [2]. The incidence of extrapulmonary tuberculosis tends to increase when the cellular immune response is compromised, as in HIV infection [11]. However, neither HIV infection nor any other immunosuppressive factor was found in our patient. Tuberculosis of the sternoclavicular joint is difficult to diagnose, which may delay treatment. Osteoarticular tuberculosis is generally a pauci-bacillary disease: a TST test can be negative in many cases, and most patients also have normal radiographs. Histology and biopsy culture are the gold standards for diagnosis and are positive in over 90% of cases. The GeneXpert test is a good tool because it can detect even traces of *M. tuberculosis* DNA. In the case of the sternoclavicular joint, CT scans can be useful in visualizing signs of arthritis with soft tissue involvement. The presence of calcified abscesses in the paravertebral and psoas muscles is almost pathognomonic for tuberculosis. Radiologic and imaging modalities are complementary, with MRI being the best technique for early detection and diagnosis of tuberculosis of the sternoclavicular joint [11]. The multifocal nature of our patient's tuberculosis may be explained by the delay in seeking health care. This delay is common in tropical and African settings. Patients often go to traditional practitioners first. This delay is compounded by delays in diagnosis due to lack of technical facilities and delays in access to treatment. These delays can alter prognosis [4]. Our patient's vital prognosis was guarded, mainly due to the pleuropulmonary involvement.

## Conclusion

Multifocal tuberculosis is a severe form of the disease that usually affects immunocompromised patients who already have lung involvement. It is rare in sickle cell patients. It should not be overlooked. Sternoclavicular localization is exceptional. Sickle cell patients should be systematically screened for tuberculosis dissemination to ensure better management.

## Patient's consent

The patient's consent was obtained for the publication of this article.

## Authors’ contributions

Bayala Yannick Laurent Tchenadoyo: writing, design, discussion

Ayouba Tinni Ismael: design, Discussion Kaboré Fulgence, Bonkoungou Marcellin: validation

Zabsonré/Tiendrebeogo Wendlassida Joëlle Stéphanie, Ouedraogo Dieu-Donné: supervision

## Declaration of interest

The authors declare no conflict of interest.
